# Zymotic Diseases—Typhus and Typhoid Fevers, Diagnosis of

**Published:** 1849-09

**Authors:** 


### SELECTIONS

FROM

### AMERICAN AND FOREIGN JOURNALS.

---

*Zymotic Diseases—Typhus and Typhoid Fevers, Diagnosis of.*—The readers of the "Half-yearly Abstract" have, at various times, been made acquainted with the discussions at home and abroad, upon the identity of typhus and typhoid fever. In addition to the opinions mentioned in former volumes, we shall here adduce those of two of the latest writers on the subject, Dr. Wood and Dr. Wilshire.

Dr. Wood, whose opportunities of witnessing the two forms of fever have been such as are afforded only in America, where they both rage with nearly equal intensity, expresses himself as follows on the means of distinguishing them :

"Typhus fever less frequently commences insensibly than enteric (typhoid), and is upon the average of shorter duration. Instead of diarrhea, or the susceptibility to purgatives, which attend the latter disease, there is usually constipation ; and the fecal discharges are darker and more offensive. Hemorrhage from the bowels, which is not infrequent in the advanced stages of enteric fever, seldom occurs in typhus. In this complaint, epistaxis at the commencement is less frequent, there is more stupor, and a darker color of the face, more turbidness of the conjunctiva, and greater debility. The eruption in typhus also differs from that of enteric fever. It generally commences earlier, is not elevated, is of a darker hue, does not so readily disappear under pressure, is much more abundant, and, instead of being confined to the abdomen and chest, is diffused over almost the whole body. In typhus, the abdomen is often flat, and perfectly free from tympanitis ; which is never the case in enteric fever.

The anatomical characters of the two fevers are very different. The peculiar disease of the glands of Peyer, and of the mesenteric glands, so constantly present in enteric fever, is never found in typhus, or so seldom, as to lead to the suspicion of an intermixture of diseases when it does occur. The spleen is less frequently enlarged and softened in typhus.

Enteric fever almost never attacks the old, who are frequent victims of typhus. The former disease is endemic in various countries, arises here and there without obvious cause, and, if ever contagious, is very feebly so ; while typhus seldom occurs in isolated cases, is always contagious, and often epidemic."

Nevertheless, the author admits there are cases of a mingled character, in which the elements of the two fevers may be supposed to co-exist.

The observations of Dr. Wilshire, which occur in the course of his valuable lectures on the diseases of infancy, are recapitulated briefly in the following tabular arrangement :

| *Typhoid Fever, Adynamic Fever, Asthenic Fever, Low Fever, &c., of France and Great Britain, &c.* | *True Typus, Contagious Typhus, True Maculated Fever of Great Britain, &c. &c.* |
|---|---|
| NOT INFECTIOUS. | EMINENTLY INFECTIOUS. |
| Cutaneous eruption often wanting; does not appear so early ; more of the nature of petechia; does not disappear under pressure—no true exanthema. | Cutaneous eruption a true exanthema—differing from petechia, which may accompany it—former of a reddish pink color, disappear under pressure, soon to return on removal of it —sometimes dark, like measles, and not removable by pressure. |
| Cerebral symptoms not appearing early—not so frequent. | Early delirium, stupor, or typhomania. |
| Course prolonged, fifteen to thirty days—relapses common. | Period of crisis about fourteenth day—relapses unfrequent. |
| Not common before fifteen years of age, nor after fifty. | Children often attacked when the disease is epidemic. |
| Attacks those predisposed to a febrile affection by exposure, vicissitudes, or is sporadic. | Generally presents itself as an epidemic, and may attack any one coming within its sphere. |
| May occur under the milder grades of the causes of typhus, or entirely without them—is sporadic. | Caused by the same—by famine as the predisposing cause; epidemic. |
| Derangement of alimentary canal constant—important alterations connected with the intestinal follicles found—spleen large—intestinal perforation frequent. | Post mortem results often negative, and when lesions are present, they bear no comparison with the severity and rapidity of production of those of typhoid fever. |

*Typhus Fever, Exanthematous Nature of.*—Contrary to the opinions expressed by the writer last mentioned, Dr. Williman has endeavored to prove that typhoid fever is a true exanthema, and as such should be placed in the same catagory with other eruptive fevers. He bases his opinion upon their similarity in the fact of incubation, in the presence of a distinct eruption, in its prevalence consentaneously with other eruptive diseases, and, like them, in the subjects of it becoming exempt from a second attack. The analogy is further exemplified in a comparison of their respective pathological lesions.—*Ranking's Abstract.*

